# Post-stroke treatment with argon preserved neurons and attenuated microglia/macrophage activation long-termly in a rat model of transient middle cerebral artery occlusion (tMCAO)

**DOI:** 10.1038/s41598-021-04666-x

**Published:** 2022-01-13

**Authors:** Jingjin Liu, Michael Veldeman, Anke Höllig, Kay Nolte, Lisa Liebenstund, Antje Willuweit, Karl-Josef Langen, Rolf Rossaint, Mark Coburn

**Affiliations:** 1 Department of Anesthesiology, University Hospital RWTH Aachen, Aachen, Germany; 2 Department of Neurosurgery, University Hospital RWTH Aachen, Aachen, Germany; 3 Department of Neuropathology, University RWTH Aachen, Aachen, Germany; 4 Institute of Neuroscience and Medicine (INM-4), Forschungszentrum Jülich, Jülich, Germany; 5 Department of Nuclear Medicine, RWTH University Hospital, Aachen, Germany; 6 Department of Anesthesiology and Intensive Care Medicine, University Hospital Bonn, Bonn, Germany

**Keywords:** Therapeutics, Stroke

## Abstract

In a previous study from our group, argon has shown to significantly attenuate brain injury, reduce brain inflammation and enhance M_2_ microglia/macrophage polarization until 7 days after ischemic stroke. However, the long-term effects of argon have not been reported thus far. In the present study, we analyzed the underlying neuroprotective effects and potential mechanisms of argon, up to 30 days after ischemic stroke. Argon administration with a 3 h delay after stroke onset and 1 h after reperfusion demonstrated long-term neuroprotective effect by preserving the neurons at the ischemic boundary zone 30 days after stroke. Furthermore, the excessive microglia/macrophage activation in rat brain was reduced by argon treatment 30 days after ischemic insult. However, long-lasting neurological improvement was not detectable. More sensorimotor functional measures, age- and disease-related models, as well as further histological and molecular biological analyses will be needed to extend the understanding of argon’s neuroprotective effects and mechanism of action after ischemic stroke.

## Introduction

Stroke has become one of the most common causes of death as well as a leading cause of major disability worldwide^1^, of which 87% are ischemic in nature^2^. So far, timely reperfusion achieved by intravenous tissue plasminogen activator (tPA) or endovascular thrombectomy remains the only effective intervention for acute ischemic stroke patients^3,4^. However, patients who are eligible for the treatment are quite limited^5,6^. Access to the treatment is also limited by resources^7^. Likewise, the intervention has considerable shortcomings, such as symptomatic hemorrhagic transformation, which is a devastating complication of intravenous thrombolysis treatment and is associated with high mortality^8,9^. Moreover, although reperfusion within a certain time window can reduce infarct size and improve clinical outcome, cerebral reperfusion injury can occur and exacerbate the brain injury^10,11^. Taken together, stroke as a major public health issue requires urgent development of new effective therapeutic strategies.

In recent years, argon has proven to be an effective neuroprotectant in an array of in vivo and in vitro models^7,11–19^. Argon is inexpensive and easy to transport. It does not have anesthetic properties at the concentrations used, therefore would not confound neurological assessment if used in a pre-hospital setting. Furthermore, ventilation with argon appears to be safe in pigs and preliminary human trials^20^. In the context of cerebral ischemia injury, argon has shown to be beneficial after both transient and permanent ischemic stroke insults^7,11,12^. Meanwhile, evidence from animal studies indicates that argon could be neuroprotective not only in a pre-hospital setting but also during later treatment in stroke unit. In a recent study, we demonstrated that argon administration with a 3 h delay after stroke onset and 1 h after reperfusion significantly alleviated neurological deficit within the first week and alleviated neuronal damage in the ischemic boundary zone 7 days after stroke. Moreover, argon reduced the excessive microglia/macrophage activation and promoted the switch of microglia/macrophage polarization towards the anti-inflammatory M_2_ phenotype^7^. However, the long-term effects of argon after cerebral ischemia injury have not been reported so far. In the present study, we further investigate the underlying neuroprotective effects of argon up to 30 days after stroke insult in accordance with the Stroke Therapy Academic Industry Roundtable recommendations^21^. Meanwhile, the long-term influence of argon on microglia/macrophage activation and polarization will also be explored.

## Results

**Figure 1:**
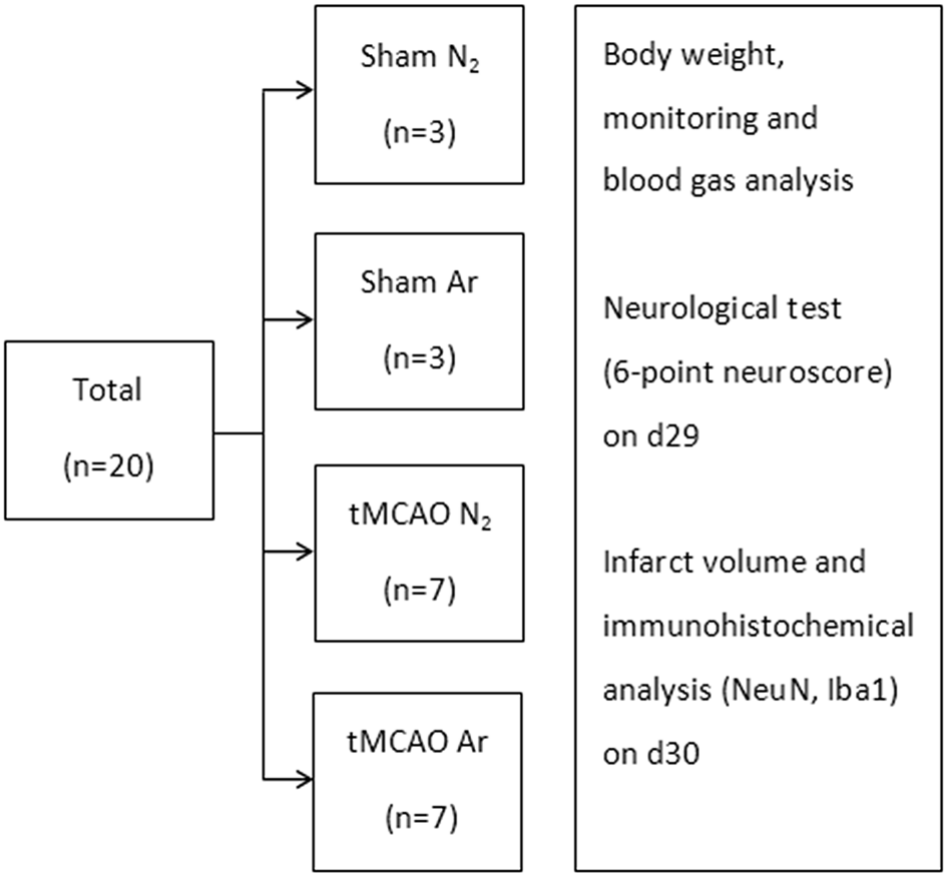
Flowchart of animal enrollment and experimental design.

All animals survived until being euthanized. Thus, the data from a total of 20 animals (n = 7 for tMCAO Ar and tMCAO N_2_ groups, n = 3 for sham Ar and sham N_2_ groups) underwent final statistical analyses for left and right rCBF, MAP, HR, blood gas analysis, baseline body weight, neuroscore, infarct volume measurement and immunohistochemical analysis. The number of animals enrolled in each group as well as the subsequent outcome measures were listed in Fig. 1.

### Monitoring, blood gas analysis and body weight

**Figure 2:**
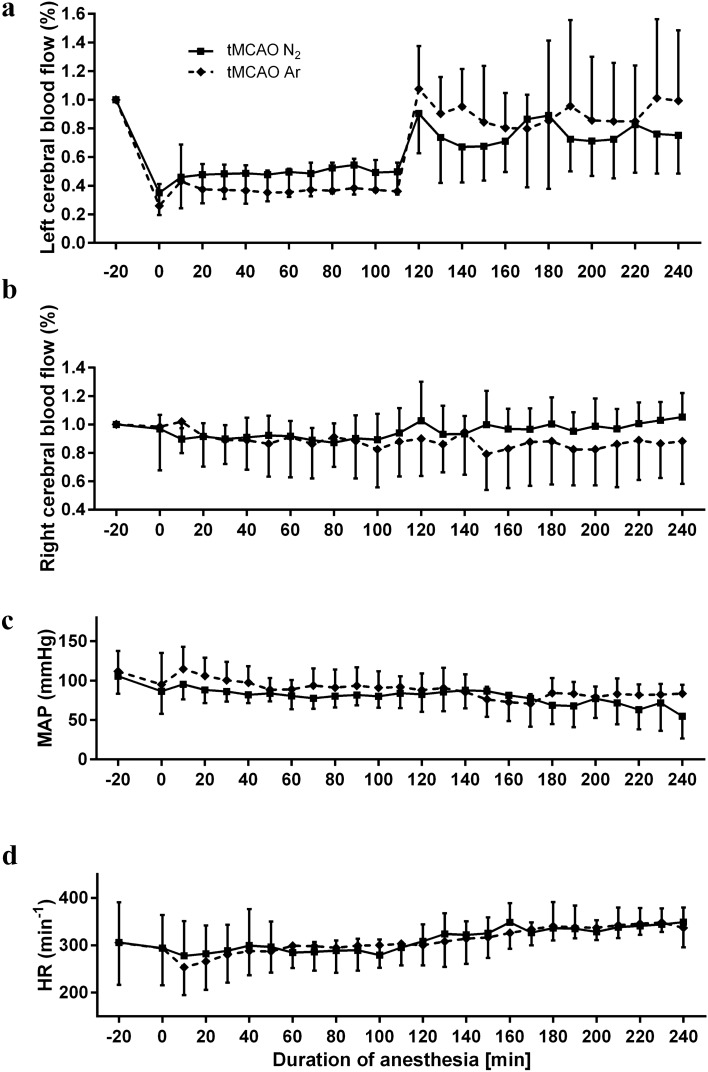
Left regional cerebral blood flow (rCBF) (**a**) and right rCBF (**b**) as percentage of baseline value; mean arterial blood pressure (MAP) in mmHg (**c**); heart rate (HR) in beats per minute (**d**). The courses of left rCBF, right rCBF, MAP and HR did not differ between tMCAO Ar and tMCAO N_2_ groups. B = baseline, 5 min after tail artery catheter insertion, time point 0 = tMCAO induction, time point 120 = start of reperfusion, time point 180 = the beginning of gas application (50% Vol N_2_/50% Vol O_2_ for tMCAO N_2_ group, 50% Vol Argon/50% Vol O_2_ for tMCAO Ar group). Data were extracted in 10 min intervals. Results were represented as mean ± SD, n = 7 for tMCAO Ar and tMCAO N_2_ groups.

The regional cerebral blood flow measurement of the left middle cerebral artery showed an expected reduction from baseline level during the tMCAO procedure. At the time point of tMCAO induction, the rCBF values dropped to 22.7% ± 11.9% for tMCAO Ar group and 30.1% ± 9.3% for tMCAO N_2_ group, respectively. In the reperfusion period, the values returned to baseline level. In the meantime, the regional cerebral blood flow measurement of the right middle cerebral artery exhibited steady values during the whole surgical procedure, which ruled out the possibility of subarachnoid hemorrhage. Neither courses of left rCBF nor right rCBF showed significant difference between tMCAO Ar and tMCAO N_2_ groups (p = 0.87 for left rCBF and p = 0.55 for right rCBF) (Fig. 2a,b). Neither courses of MAP nor courses of HR showed significant difference between tMCAO Ar and tMCAO N_2_ groups (p = 0.39 for MAP and p = 0.94 for HR) (Fig. 2c,d). At time points before tMCAO, after tMCAO induction, after onset of reperfusion and after the beginning of gas application (50% Vol N_2_/50% Vol O_2_ for tMCAO N_2_ group, 50% Vol Argon/50% Vol O_2_ for tMCAO Ar group), none of the parameters of blood gas analysis (pH, pCO_2_, pO_2_, cK^+^ and cNa^+^) differed statistically between tMCAO Ar and tMCAO N_2_ groups (p = 0.06 for pH, p = 0.81 for pCO_2_, p = 0.80 for pO_2_, p = 0.82 for cK^+^ and p = 0.37 for cNa^+^) (Table 1). The baseline body weights of rats were comparable in tMCAO Ar and tMCAO N_2_ groups (336.1 ± 19.8 g v.s. 358.5 ± 33.2 g, p = 0.42).

**Table 1:** Blood gas analysis.

	Time 1	Time 2	Time 3	Time 4
**pH**				
tMCAO N_2_	7.32 (0.08)	7.35 (0.07)	7.33 (0.04)	7.38 (0.03)
tMCAO Ar	7.42 (0.10)	7.42 (0.04)	7.38 (0.04)	7.42 (0.05)
**pCO**_**2**_				
tMCAO N_2_	52.9 (7.1)	45.8 (6.0)	51.1 (4.1)	39.9 (5.1)
tMCAO Ar	48.2 (1.6)	45.2 (5.5)	49.1 (5.3)	45.5 (6.0)
**pO**_**2**_				
tMCAO N_2_	155.0 (13.6)	133.0 (8.7)	176.0 (14.3)	201.7 (6.8)
tMCAO Ar	150.0 (6.2)	163.0 (14.7)	183.7 (8.1)	163.0 (12.9)
**cK**^**+**^				
tMCAO N_2_	5.4 (0.5)	5.4 (0.7)	5.0 (0.6)	4.5 (1.0)
tMCAO Ar	5.1 (0.1)	5.1 (0.6)	5.1 (0.5)	5.1 (0.3)
**cNa**^**+**^				
tMCAO N_2_	136.3 (4.0)	132.7 (16.3)	133.3 (11.9)	142.7 (5.5)
tMCAO Ar	134.3 (9.1)	139.3 (3.1)	141.3 (1.2)	142.0 (4.4)

Results were represented as mean (SD), n = 7 for tMCAO Ar and tMCAO N_2_ groups. Time 1 = baseline, 5 min after tail artery catheter insertion, Time 2 = after tMCAO induction, Time 3 = after onset of reperfusion, Time 4 = after the beginning of gas application (50% Vol N_2_/50% Vol O_2_ for tMCAO N_2_ group, 50% Vol Argon/50% Vol O_2_ for tMCAO Ar group).

### Argon preserved neurons at the ischemic boundary zone long-termly after tMCAO

**Figure 3:**
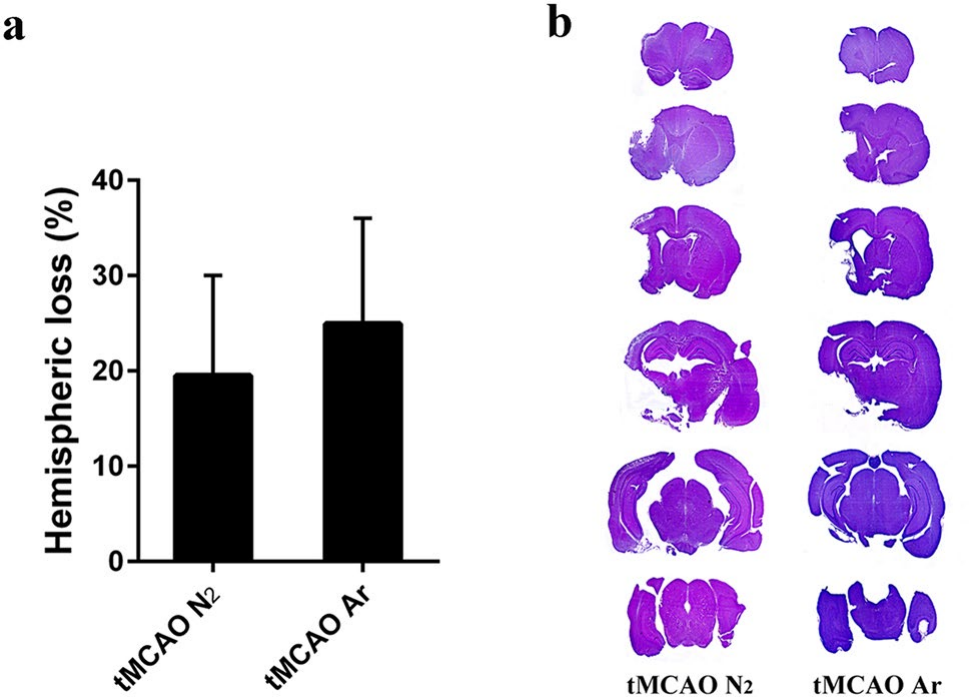
Quantification of brain infarct volume 30 days after reperfusion. Results were represented as mean ± SD, n = 7 for tMCAO Ar and tMCAO N_2_ groups.

To determine whether argon has neuroprotective effects after ischemic stroke, firstly, we measured the infarct volume 30 days after reperfusion. Quantitative analysis of the percentage of brain hemispheric loss, which reflected the originally infarcted tissue, did not show significant difference between tMCAO Ar and tMCAO N_2_ groups (p = 0.41) (Fig. 3).

**Figure 4:**
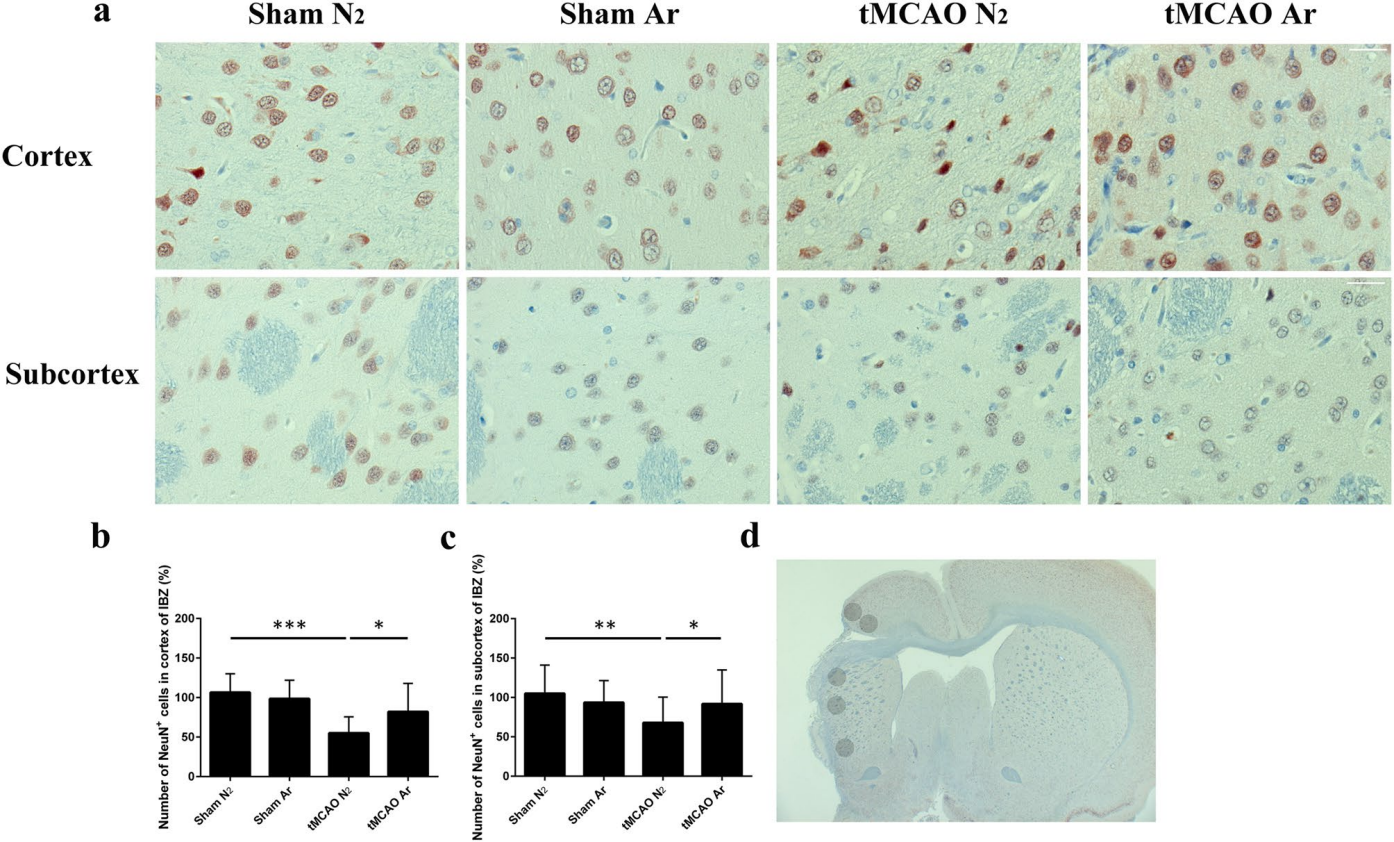
Neuronal nuclear antigen (NeuN) staining of rat brain samples. Representative images of NeuN immunohistochemistry in cortex and subcortex of ischemic boundary zone (IBZ) 30 days after reperfusion (**a**); relative number of NeuN positive cells detected in cortex (**b**) and subcortex (**c**) of IBZ; representative image of regions of interests (ROIs) for NeuN quantification (**d**). Significance was indicated with ***p < 0.001, **p < 0.01 and *p < 0.05. Argon treatment significantly increased the relative numbers of NeuN positive cells in cortex and subcortex of IBZ compared to tMCAO N_2_ group (p = 0.030 for cortex and p = 0.030 for subcortex). Results were represented as mean ± SD, n = 7 for tMCAO Ar and tMCAO N_2_ groups, n = 3 for sham Ar and sham N_2_ groups.

We analyzed subsequently the effect of argon on the survival of neurons at IBZ area 30 days after reperfusion using NeuN immunostaining. NeuN staining is robust and is widely used to detect neurons^7^. Quantitative analysis revealed that the relative numbers of NeuN positive cells in the ROIs of tMCAO N_2_ group were significantly fewer than the values of Sham N_2_ group (p = 0.00017 for cortex and p = 0.0029 for subcortex). In contrast, the relative numbers of NeuN positive cells in the ROIs of tMCAO Ar group did not differ from those of Sham N_2_ group (p = 0.51 for cortex and p = 0.63 for subcortex). When compared to tMCAO N_2_ group, the relative numbers of NeuN positive cells in the ROIs of tMCAO Ar group were significantly higher (p = 0.030 for cortex and p = 0.030 for subcortex). (Fig. 4).

### Argon suppressed microglia/macrophage activation long-termly after tMCAO

**Figure 5:**
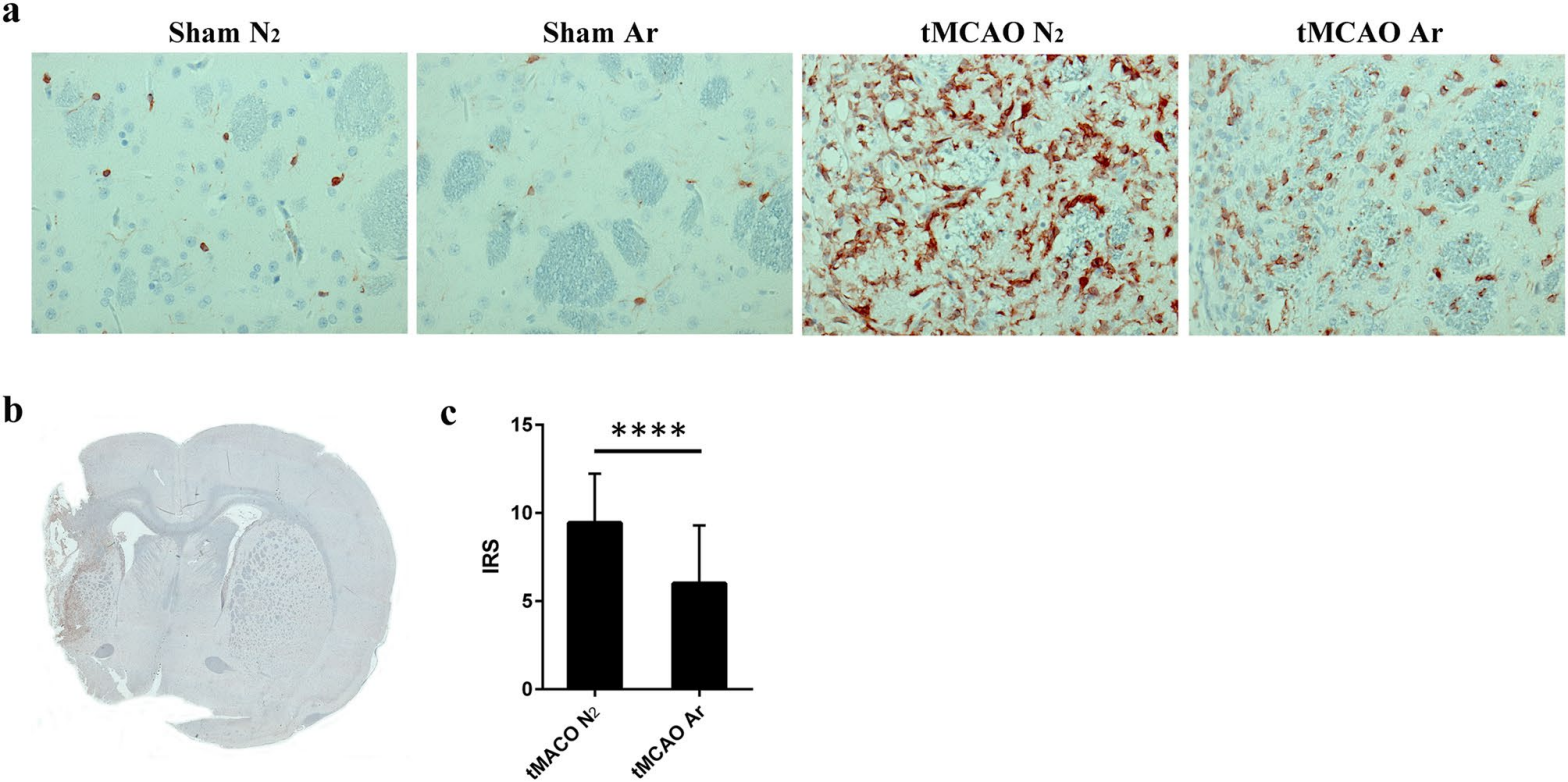
Microglia/macrophage activation with ionized calcium binding adaptor molecule 1 (Iba1) immunohistochemistry in rat brain 30 days after reperfusion. Representative images of resting microglia in sham groups and activated microglia/macrophages at IBZ in tMCAO groups 30 days after reperfusion (**a**); representative image of overall microglia/macrophage activation in rat brain sample of tMCAO N_2_ group 30 days after reperfusion (**b**); quantitative analysis of microglia/macrophage activation using immunoreactive score (IRS) 30 days after reperfusion (**c**). Significance was indicated with ****p < 0.0001. Treatment of argon significantly suppressed the microglia/macrophage activation in rat brain (p < 0.0001). Results were represented as mean ± SD, n = 7 for tMCAO Ar and tMCAO N_2_ groups.

Previous studies revealed that the microglia/macrophage activation is fully developed hours to days after ischemic stroke onset^22–24^ and declines thereafter. A similar tendency was also observed in our studies. The intensity of microglia/macrophage activation 30 days after reperfusion reduced dramatically compared to 7 days after reperfusion^7^. Notably, while three functionally and morphologically different types of microglia/macrophages were observed 7 days after ischemic stroke, namely the classical resting microglia within the non-affected areas, the intermediately activated “stellate” microglia at the IBZ and the highly activated “amoeboid” microglia/macrophages at the ischemic core^7^, only resting microglia and “stellate” microglia were found in rat brain 30 days after ischemia onset. The “amoeboid” microglia/macrophages were no longer visible as the damaged tissue was taken up by cells and a cavity formed within the ischemic core. Quantitative analysis using IRS demonstrated that argon treatment significantly suppressed microglia/macrophage activation in rat central nervous system (CNS) 30 days after reperfusion (p < 0.0001) (Fig. 5).

### Argon did not alleviate neurological deficit long-termly after tMCAO

**Figure 6:**
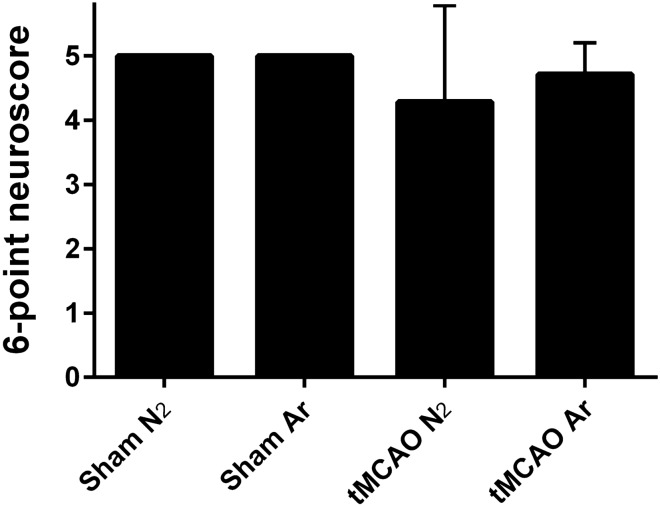
6-point neuroscore tested on d29 after reperfusion. Results were represented as mean ± SD, n = 7 for tMCAO Ar and tMCAO N_2_ groups, n = 3 for sham Ar and sham N_2_ groups.

The 6-point neuroscore on d29 did not reveal any significant difference between the four groups (p = 0.57) (Fig. 6).

## Discussion

In a previous study, we found that argon treatment 3 h after stroke onset and 1 h after reperfusion improved neurological performance during the first week after ischemic stroke, preserved the neurons of the ischemic boundary zone and alleviated the excessive microglia/macrophage activation in rat CNS 7 days after the insult^7^. In the present study, it was further demonstrated that the same treatment strategy protected the neurons and reduced the microglia/macrophage activation at the IBZ 30 days after ischemic stroke. However, the neuroscore assessed on d29 was incapable to detect any difference between different experimental settings. The findings of long-lasting neuroprotective and anti-inflammatory effects of argon after ischemic stroke strengthened the role of argon as a new promising therapeutic agent in stroke treatment. However, additional studies are needed to elucidate to what extent and under which circumstances argon will be beneficial after ischemic stroke insult.

### The long-lasting neuroprotective and anti-inflammatory effects of argon after ischemic stroke

In recent years, the neuroprotective effects of argon in context of cerebral ischemia injury have been reported in several studies using different experimental stroke models and with different therapeutic regimens^7,11,12^. When administered for 1 h until reperfusion with a 1 h delay after tMCAO induction, argon has demonstrated to reduce the infarct volume and relieve the composite adverse outcome 24 h after reperfusion^12^. Moreover, using the same therapeutic strategy as in the present study, namely with a 3 h delay after stroke onset and 1 h after reperfusion, argon has shown to significantly ameliorate neurological deficit during the first week after stroke and to promote neuronal survival in the ischemic boundary zone 7 days after reperfusion^7^. In the present study, it was further proven that argon could preserve neurons at the ischemic boundary zone until 30 days after reperfusion. However, no influence of argon on the infarct volume was detected.

Although statistical analysis didn’t reveal any difference of the course of left rCBF between tMCAO Ar and tMCAO N_2_ groups in the present study, the degree of rCBF reduction during the period of middle cerebral artery occlusion and the degree of rCBF recovery after reperfusion were not identical between two groups. Animals in tMCAO Ar group had a relative severer decrease of cerebral blood flow and a relative higher degree of reperfusion. These variations may have impact on the histological and functional outcomes in the present study.

Interestingly, Ma and colleagues^11^ found that argon inhalation for 24 h immediately after onset of permanent focal cerebral ischemia could provide neuroprotection and improve neurological outcome 7 days after the insult; however, when argon treatment was delayed for 2 h after permanent stroke induction or until after reperfusion, neurological outcome was improved, but no reduction of infarct size was detected. Similar finding with respect to the inconsistency of infarct size and functional outcome was also revealed in our previous study^7^, in which argon was administered 3 h after transient ischemic stroke onset. These findings indicate that an early start of treatment is essential to accomplish optimal protection, therefore, should be strived to achieve. On the other hand, one should be aware that the correlation between infarct size and functional impairments is at best moderate and the value of histological lesion size as a clinically relevant outcome predictor is still controversial^7,11,21,25–28^. The severity of neurological impairments also depends on the actual location of the lesion, whereas size may be less important^11,29^. Besides, the functional and/or structural reorganization of the remaining brain plays important roles after ischemic stroke too. In particular, the peri-infarct tissue is an important target for neurorepair and neuroprotective therapies^7,30^. The long-lasting neuroprotective and anti-inflammatory effects of argon at the ischemic boundary zone may be a reasonable explanation for the functional improvement after delayed argon treatment.

Inflammation is considered as a key contributor to the pathophysiology of ischemic stroke^31,32^ and microglia activation is the first step of inflammatory response in the CNS^32–34^. Accumulating evidence suggests that activated microglia after ischemic stroke insult might be a therapeutic target to limit neuronal cell death and improve clinical outcome^32,35,36^. In the present study, along with the long-lasting neuroprotective effect of argon after ischemic stroke, it was revealed that argon treatment could long-termly alleviate the excessive microglia/macrophage activation.

Excessively activated microglia/macrophages following ischemic stroke could be detrimental to neuronal survival and repair by producing proinflammatory cytokines and cytotoxic substances, influencing astrocyte activation and reactive gliosis, and affecting neurogenesis^32,37,38^. Further studies are needed to elucidate the detailed mechanisms involved in argon’s neuroprotective and anti-inflammatory effects after ischemic stroke.

Strikingly, accumulating evidence reveals that microglia/macrophages may have a dual role in ischemic stroke^32,37,39^. Despite the traditional deleterious role, studies have shown that microglia could also contribute to tissue repair and remodeling by clearing up debris and producing anti-inflammatory cytokines and growth factors, and could be beneficial for the functional recovery after cerebral ischemia^32,40–42^. Microglia were demonstrated to be supportive of neurogenesis under certain circumstances^32^. The distinct role of microglia/macrophages after brain injury may be attributed to different polarization under different cellular contexts and pathological stages^32,37,39^. Therefore, exclusively suppressing microglia activation may not be a suitable therapeutic strategy for ischemic brain injury, as the beneficial role of microglia/macrophages might be compromised. Therapeutic approaches should aim at modulating the activation of microglia and drive microglia polarization to a protective phenotype^32,39^. Given that argon could long-termly suppress the microglia/macrophage activation and meanwhile promote the switch of microglia/macrophage polarization towards the anti-inflammatory M_2_ phenotype^7^, it may serve as a new promising therapeutic approach in ischemic stroke treatment.

### Functional outcomes

*Wistar* rats were chosen as the experimental subject in the present study because studies showed that different rat strains display different behavioral and histological outcomes after experimental ischemia^43–45^ and that tMCAO induces larger ischemic lesions and formation of edema in *Wistar* rats^45^. It is surprising that no statistical difference of the 6-point neuroscore was found between animals who received tMCAO and the sham animals, although a massive decease of the rCBF value was achieved at the tMCAO induction (the left rCBF values deceased to 23.8% for tMCAO Ar group and 30.7% for tMCAO N_2_ group) and a considerable hemispheric loss was demonstrated 30 days after stroke (24.9% for tMCAO Ar group and 19.5% for tMCAO N_2_ group). Due to the spontaneous recovery after stroke event, a statistical significance of the functional test was undetectable between argon and placebo treatment.

Spontaneous recovery after stroke has been reported to be common for many functional outcome measures^21,46,47^. A sociodemographic study with regard to the prevalence of poststroke cognitive impairment revealed an overall prevalence ratio of long-lasting cognitive impairment to be 22%^48^. Moreover, in most of the animal studies, cerebral ischemia was induced in young healthy animals, in which the spontaneous recovery may be more likely. Whereas stroke in humans occurs as a result of the natural progression of underlying diseases or risk factors, such as aging, hypertension and diabetes^49^. Suenaga et al.^50^ demonstrated that aged rodents exhibit significantly severer long-term sensorimotor and cognitive deficits combined with significantly larger brain infarct size. In humans, it has also been stated that the cognitive impairment rate in stroke survivors was strongly associated with age^48^. Taken together, considering the potential possibility of spontaneous recovery after stroke in different functional outcomes, a battery of neurological and behavioral assessments would be beneficial to draw a panorama of the functional protective effect of potential treatments^47,51^. Related to the present study, more behavioral measures with respect to different aspects of sensorimotor function, such as Rotarod, Cylinder test, Catwalk test and Forelimb placing, are needed in future^46,47^. Further, studies performed in age-related models or disease-related models may be of great interest and would extend the understanding of argon’s neuroprotective effect after stroke.

### Limitations and prospective

The design of a 3 h-delayed argon administration after stroke onset is more relevant to clinical practice^3^ and would provide valuable data to guide the development of new therapeutic strategies for patients. In addition, continuous perioperative monitoring, especially the bilateral rCBF assessment, assured experimental quality and eliminate falsification of the experimental results by including undesired pathologies^51^. For the first time, to the best of our knowledge, the long-term effects of argon after ischemic stroke were explored. The findings with regard to the long-lasting neuroprotective and anti-inflammatory effects of argon after stroke could deepen the understanding of the treatment strategy. However, several limitations exist in the present study. Further studies are needed to confirm and extend the findings of the present study.

Although appropriate sample size was calculated based on the determined primary outcome, namely the 6-point neuroscore on d29, considering the possibility of spontaneous recovery, a much larger sample size is highly advisable in order to detect the potential benefit of the treatment. Age-related and disease-related models should be taken into account in future studies. Meanwhile, the functional tests being used should be chosen carefully and a battery of neurological and behavioral assessments would be beneficial to reveal the potential protective property of the therapeutic regimen. Computer-assisted and automated systems may offer an additional objective evaluation^51,52^.

Adequate observation time points for assessing long-term functional performance are needed to form an overall perspective of the potential treatment effect. On the one hand, the functional performance after stroke may be fluctuant^7,47,53^. On the other hand, given that in adulthood every day of a laboratory rat is approximately equivalent to 34.8 human days^54^, an improvement even within the first two or three weeks after experimental ischemia onset would be of great importance for clinical practice^21^.

Except for microglia/macrophage activation and polarization, more functional and/or structural alterations, including astrocyte reactivity, neurogenesis, blood–brain barrier permeability, cerebral vasoconstriction, synaptic plasticity, axonal remodeling and angiogenesis at the ischemic boundary zone, even within the non-affected hemisphere, are of importance to uncover the panorama of argon’s neuroprotective effects after ischemic stroke^7^. Furthermore, molecular pathways which are involved in argon’s neuroprotective and anti-inflammatory effects need to be further elucidated.

50% Vol argon was used in the present study based on a previous in vitro study from our group^14^, which demonstrated that 50% argon offers a maximum neuroprotective effect compared to 25% and 74% argon. Importantly, human data from a small cohort of healthy volunteers (n = 4) showed that exposure to 50% argon is safe for up to 6 days^55^. Besides, from a clinically relevant consideration, 50% of argon allows a higher inspiratory oxygen concentration for patients in demand^14^. However, studies are needed to further assess the optimal concentration, timing and duration of argon application in in-vivo experimental stroke models.

Current evidence shows significant protective effect of argon after both transient and permanent ischemic stroke insults^7,11,12^. However, detailed experimental setups differ between studies. Considering the heterogeneity of human stroke, studies including both disease subgroups and concerning the similarities and differences in their protective mechanisms would be of great interest.

## Conclusions

In the present study, argon administration with a 3 h delay after stroke onset and 1 h after reperfusion demonstrated long-term neuroprotective and anti-inflammatory effects in the rat brain. However, long-lasting neurological improvement was not detectable. More sensorimotor functional measures, age- and disease-related models, as well as further histological and molecular biological analyses will be needed to extend the understanding of argon’s neuroprotective effects and mechanisms after ischemic stroke. Given that argon is easy to apply (via face mask) and is lack of apparent toxicity^11,20^, it is neuroprotective and suppresses microglia/macrophage activation while promotes the switch of microglia/macrophage polarization towards the anti-inflammatory M_2_ phenotype^7^, it may serve as a new promising therapeutic approach for ischemic stroke patients.

## Materials and methods

### Animals and treatment groups

Animals were housed and assigned to groups as described before^7^. A total of 32 male *Wistar* rats (body weight 300–400 g; Charles River, Sulzfeld, Germany) were housed for at least 1 week before surgery with free access to food and water on a 12-h light/dark cycle. A parallel design was applied to the present study with the allocation ratio of 1:1. Animals were randomly assigned by drawing lots. Animals were sequentially numbered to ensure the allocation concealment. The assignments were enclosed in sealed envelopes and were not accessible to the researchers who were responsible for animal care as well as behavioral and histological assessments.

### tMCAO procedure

The tMCAO procedure was performed as described before^7^. Anesthesia was induced by intraperitoneal injection of midazolam (2 mg/kg) (Ratiopharm, Ulm, Germany), medetomidine (0.15 mg/kg) (Zoetis, Florham Park, NJ), and fentanyl (0.005 mg/kg) (Rotexmedica, Trittau, Germany), and maintained by hourly intraperitoneal application of 0.03–0.05 ml of this anesthetics combination. Animals were intubated and ventilated with 50% Vol N_2_/50% Vol O_2_. Blood gas analysis was conducted for each animal to insure proper ventilation, at: 5 min after tail artery catheter insertion, after tMCAO induction, after onset of reperfusion and after the beginning of gas application. A polyethylene catheter was inserted into the tail artery to measure blood pressure and to take blood samples for blood gas analysis. Electrocardiographic needle electrodes were placed for continuous heart rate monitoring.

Briefly, a silicone-coated 4–0 nylon monofilament was introduced via left common carotid artery into the internal carotid artery, and advanced until resistance was felt and the cerebral blood flow measurement of the left side showed a sufficient drop. Reperfusion was accomplished by withdrawal of the filament 2 h after tMCAO induction. One hour after reperfusion, animals received either 50% Vol Argon/50% Vol O_2_ (Air Liquide, Paris, France) or 50% Vol N_2_/50% Vol O_2_ for 1 h. During the entire surgical procedure and treatment, body temperature was maintained at 37–37.5 °C through a feedback-controlled heating pad (Physitemp Instruments, Clifton, NJ). Analgesic treatment (Flunixin, 1 mg/kg s.c.) was carried out daily from the day of surgery till d3.

### Assessment of regional cerebral blood flow (rCBF)

The rCBF assessment was performed as described before^7^. The rCBF assessment over the left and right middle cerebral artery was performed by laser Doppler flowmeter (Moor Instruments, Axminster, Devon, United Kingdom) during the entire tMCAO procedure and treatment to insure appropriate middle cerebral artery occlusion and reperfusion. The laser Doppler probes were placed on the animal’s skull approximately 1 mm posterior to the bregma and 5 mm lateral to the midline. Baseline measurement was taken 5 min after tail artery catheter insertion.

### 6-Point neuroscore

Neurological function was examined by a blinded investigator using the 6-point neuroscore as reported previously^7,12^. The neuroscore was evaluated on d29. The 6-point neuroscore was graded in six levels from 0 to 5: 5 = normal motor function, no neurologic deficit; 4 = flexion of torso and contralateral forelimb when lifted by the tail; 3 = decreased resistance to lateral push without circling; 2 = circling to the contralateral side against resistance when tugged by the tail on a flat surface; 1 = circling spontaneously to the contralateral side; 0 = no spontaneous motor activity, loss of walking or righting reflex.

### Tissue sampling

The tissue sampling was performed as described before^7^. Rats were euthanized on d30 by terminal anesthesia. After that, brains were removed and immediately fixed in 4% paraformaldehyde. Forty-eight hours later, rat brains were sectioned into 2 mm coronal blocks, and in total, six blocks per animal were embedded in paraffin. A 2-µm section from the anterior side of each block was collected for hematoxylin–eosin staining and the following infarct volume measurement. Sections from the 4th block (1 mm to -1 mm in the coronal plane from Bregma) were used for other stainings (see below).

### Infarct volume measurement

The infarct volume measurement was performed as described before^7^ and was modified to fit the present experimental design. Sections were stained with routine hematoxylin–eosin and were visualized and photographed with the EVOS FL Auto Imaging System (Life Technologies, Carlsbad, California, United States) using a 10 × objective. Images were analyzed using ImageJ 1.46r (https://imagej.nih.gov/ij). The infarct volume was calculated by an indirect method of subtracting the non-lesioned volume of the ipsilateral hemisphere from the non-lesioned volume of the contralateral hemisphere, and was normalized to the volume of the contralateral hemisphere of the same section.

### Immunohistochemistry

The immunohistochemical stainings and subsequent analyses were performed as described before^7^ and were modified to fit the present experimental design. 2-µm sections were cut from paraffin-embedded brain blocks and were placed on silane-coated slides. Sections were dewaxed, rehydrated and heated in citrate buffer for antigen retrieval. Nonspecific binding was blocked by incubating sections in phosphate buffered saline containing either 1% normal goat serum or 1% bovine serum albumin (BSA). The following primary antibodies were used: mouse anti-neuronal nuclear antigen (NeuN) (1:200; Merck Millipore, Billerica, MA) and rabbit anti-ionized calcium binding adaptor molecule 1 (Iba1) (1:500; Wako Chemicals, Neuss, Germany). Stained sections were photographed with the Axioplan microscope (ZEISS, Oberkochen, Germany) at 40 × objective using Zen 2 blue edition (https://www.zeiss.com/microscopy/int/products/microscope-software/zen.html#). For NeuN immunostaining, regions of interests (ROIs) were set to the cortex and subcortex of ischemic boundary zone (IBZ). Nuclei were counted in two images from cortex and three images from subcortex of IBZ. Values were expressed as relative values in relation to the number of neurons counted in images collected from identical locations in the contralateral hemisphere of the same slice. For Iba1 immunostaining, three images of IBZ were used for analysis. Quantification of the results was performed by using the immunoreactive score (IRS) according to Remmele and Stegner, which considers both the staining intensity and the number of stained cells^56^.

### Quantification and statistical analysis

The statistical analysis was performed as described before^7^ and was modified to fit the present experimental design. The 6-point neuroscore on d29 was determined as the primary outcome. Sample size was calculated based on our previous study^7^. The calculation was performed using nQuery Advisor + nTerim 4.0 (Statistical Solutions, Saugus, MA, USA). The portal used for one-way analysis of variance (ANOVA) data was selected. The number of groups was set to 4. An average difference of 0.7 between groups was expected, and the standard deviation was assumed to be 1.2. The significance was set to 5% and the statistical power to 80%. Thus, the minimum sample size was defined as n = 7. All data were expressed as mean ± SD. Normality of the data was tested by Shapiro–Wilk test and homogeneity of variance was tested by Levene’s test. Independent t-Test was used to perform the comparison between two groups (tMCAO Ar v.s. tMCAO N_2_ group). One-Way ANOVA was applied to assess comparisons between four groups, if necessary, Bonferroni post hoc test was used for following multiple comparisons. Courses of left and right rCBF, mean arterial blood pressure (MAP) and heart rate (HR), and blood gas analysis were compared using repeated measures ANOVA. All calculations were performed using SPSS 23.0 (IBM, Chicago, IL). p < 0.05 was considered statistically significant.

### Ethics approval and consent to participate

The research protocol and animal care procedures of this study were approved by the State Office for Nature, Environment and Consumer Protection (AZ 84–02.04.2013.A418, Landesamt für Natur, Umwelt und Verbraucherschutz Nordrhein-Westfalen). All experiments were performed in accordance with the German legislation governing animal studies (Tierschutzgesetz, Tierschutz-Versuchstierverordnung) and the ARRIVE guidelines 2.0^57^.

## Data Availability

All data generated or analysed during this study are included in this published article.

## Abbreviations

tMCAO: Transient middle cerebral artery occlusion
rCBF: Regional cerebral blood flow
BSA: Bovine serum albumin
NeuN: Neuronal nuclear antigen
Iba1: Ionized calcium binding adaptor molecule 1
ROIs: Regions of interests
IBZ: Ischemic boundary zone
IRS: Immunoreactive score
ANOVA: Analysis of variance
MAP: Mean arterial blood pressure
HR: Heart rate
CNS: Central nervous system
